# Antioxidant, Antidiabetic, Anti-Obesity, and Anti-Inflammatory Activity of Tomato-Based Functional Snack Bars Enriched with Pea and RuBisCO Proteins

**DOI:** 10.3390/foods14193340

**Published:** 2025-09-26

**Authors:** Elena Tomassi, Morena Gabriele, Agnese Sgalippa, Muhammed Rasim Gul, Ozan Tas, Mecit Halil Oztop, Laura Pucci

**Affiliations:** 1 Institute of Agricultural Biology and Biotechnology, National Research Council, 56124 Pisa, Italy; elena.tomassi@ibba.cnr.it (E.T.); morena.gabriele@ibba.cnr.it (M.G.); agnese.sgalippa@outlook.com (A.S.); 2 Department of Food Engineering, Middle East Technical University, Ankara 06800, Turkey; rasim.gul@metu.edu.tr (M.R.G.); ozantas@metu.edu.tr (O.T.); mecit@metu.edu.tr (M.H.O.)

**Keywords:** snack bars, antioxidant capacity, plant proteins, digestibility

## Abstract

Snack bars are convenient, ready-to-eat foods with various natural ingredients and may serve as functional foods, offering bioactive phytochemicals. In this study, tomato-based snack bars enriched in plant proteins were evaluated for their antioxidant, antidiabetic, anti-obesity, and anti-inflammatory properties by in vitro test, comparing different protein sources (pea and RuBisCO) and drying methods (microwave vacuum and oven). The rubisco bars exhibited the highest levels of polyphenols (10.12 ± 0.27 mg GAE/g) and flavonoids (5.65 ± 0.47 mg CE/g), and demonstrated superior antioxidant capacity in DPPH, ORAC, and FRAP assays, particularly when microwaved. Rubisco bars also exhibited better inhibition activity of dipeptidyl-peptidase IV and pancreatic lipase, suggesting potential antidiabetic and anti-obesity effects. In contrast, pea bars displayed notable anti-inflammatory effects by reducing tumor necrosis factor (TNF)-α-induced cyclooxygenase-2 (COX-2) expression in intestinal cells. Both protein types were digestible, though rubisco bars released more peptides during simulated gastrointestinal digestion. While these in vitro findings provide insights into the functional potential of tomato-based snack bars, further studies, including in vivo investigations, are required to confirm their health-promoting effects and to evaluate physiologically relevant doses. Overall, these findings highlight the potential of tomato-based snack bars as sustainable, nutrient-rich functional foods with potential health-promoting properties.

## 1. Introduction

Functional foods are designed to provide specific health benefits beyond basic nutrition, primarily due to the presence of bioactive compounds that help reduce the risk of various chronic diseases [1]. The development of such foods requires careful formulation of ingredients and the selection of processing methods that maximize both the retention and the bioavailability of these beneficial compounds.

Processing plays a critical role in determining the nutritional and functional quality of foods. Conventional thermal treatments are widely applied in the food industry, but they can enhance or degrade bioactive compounds depending on applied conditions [2,3]. More recently, alternative methods such as microwave (MW) drying have attracted increasing attention for their efficiency and sustainability. Compared to conventional approaches, microwave vacuum drying has emerged as a promising alternative, offering faster dehydration and improved retention of bioactive compounds while reducing energy consumption [4,5]. This technology has already shown potential benefits in fruits and vegetables [6], yet its impact on complex matrices such as snack bars is still underexplored.

Snack bars have become a popular category of convenient, ready-to-eat foods, valued for their compact size and balanced macronutrient content. Their versatility allows for the incorporation of diverse natural ingredients, making them promising vehicles for delivering functional bioactive compounds [7,8]. Given the rising consumer demand for health-promoting snacks, the global functional snack market is rapidly expanding, driven by growing awareness of diet-related chronic diseases and the need for practical, nutrient-dense options.

The Mediterranean diet, widely recognized for its health benefits, provides a valuable framework for the design of such products. Ingredients of this diet—such as tomatoes, olives, and various herbs—are rich sources of antioxidants and bioactives like lycopene, flavonoids, vitamins, and minerals, which have been linked to both protective effects against cancer, neurodegenerative disorders, and diabetes, and maintenance of gut health [9,10,11,12].

Building upon these principles, the Middle East Technical University (METU), as part of the FunTomP (Functionalized Tomato Product) project, has developed tomato-based snack bars enriched with tomato peel powder, olive powder, and plant-derived proteins. Two protein sources were selected for enrichment: pea protein, known for its digestibility, emulsifying capacity, and bioactive peptides with antioxidant and antihypertensive effects [13]; and RuBisCO (ribulose-1,5-bisphosphate carboxylase/oxygenase), a leaf protein extracted from sugar beet leaves, a sustainable by-product common in Europe and North Africa [14]. This latter is gaining growing interest as a novel, sustainable protein source with high nutritional quality and unique functional properties, including antioxidant activity and high digestibility [15,16]. However, its application in food systems is still limited, and little is known about its contribution to the functional profile of complex matrices such as snack bars.

Although functional foods, drying technologies, and protein fortification have been widely studied, the combined effect of protein source and drying method on the functional properties of tomato-based snack bars remains poorly understood. Therefore, the aim of this study was to evaluate the effect of two protein sources (pea and RuBisCO) and two drying methods (microwave vacuum and conventional oven) on the antioxidant, antidiabetic, anti-obesity, and anti-inflammatory properties of tomato-based snack bars.

## 2. Materials and Methods

### 2.1. Chemicals and Reagents

The Folin–Ciocalteu reagent, sodium carbonate (Na_2_CO_3_), sodium hydroxide (NaOH), gallic acid, catechin, phosphate buffer (PB), acetate buffer, 6-hydroxy-2,5,7,8-tetramethylchromane-2-carboxylic acid (Trolox), 2,2-diphenyl-1-picrylhydrazyl (DPPH), 2,4,6-Tri(2-pyridyl)-s-triazine (TPTZ), sodium nitrite (NaNO_2_), aluminum chloride (AlCl_3_), ferric chloride hexahydrate (FeCl_3_-6H_2_O), ferrous sulfate heptahydrate (FeSO_4_-7H_2_O), 2,2′-azobis(2-amidinopropane) dihydrochloride (AAPH), fluorescein, porcine lipase type II enzyme, *p*-Nitrophenyl butyrate (PNTB), Bradford reagent, orlistat, trichloroacetic acid (TCA), 2′,7′-dichlorofluorescein diacetate (DCFH-DA), and 3-(4,5-Dimethyl-2-thiazolyl)-2,5-diphenyl-2H-tetrazolium Bromide (MTT) were purchased from Sigma-Aldrich (Saint Louis, MO, USA) as well as ethanol from VWR (Radnor, PA, USA).

For tomato snack bar formulations, fresh tomatoes were purchased from Kraft Heinz Food (Balıkesir, Turkey). Pea protein isolate (PPI) was obtained from Vegrano (Başakşehir, Istanbul, Turkey). RuBisCO protein was obtained from sugar beet leaves through alkaline extraction based on the study of Akyüz et al. [17]. Olive powder was prepared by freeze-drying after homogenizing green olives [18]. Salt, basil, mint, thyme, and red pepper powders were purchased from a local market. Low-methoxylated pectin (LMP, 27%) was purchased from Cargill (Balıkesir, Turkey).

### 2.2. Bar Samples Composition and Extraction

The bar samples, developed and supplied by Middle East Technical University (METU, Ankara, Turkey), were initially formulated and produced based on the study of Gul et al. [3], which extensively explored different snack bar formulations focusing on enhancing bio-functional properties (e.g., lycopene content), texture (e.g., hardness, gumminess, chewiness), and sensory characteristics, while also aiming to reduce raw material usage. Following this study, a response surface methodology (RSM) optimization was conducted to identify the most promising formulations, and the final bar samples used in the present work were selected based on these optimized conditions. Table 1 shows the ingredients and process of creating tomato bar samples.

Four different snack bar formulations were prepared accordingly, as detailed below:

Sample 1-Pea-protein-added microwave-vacuum-dried tomato snack bars.
  Sample 2-RuBisCO-protein-added microwave-vacuum-dried tomato snack bars.
  Sample 3-Pea-protein-added conventionally dried tomato snack bars.
  Sample 4-RuBisCO-protein-added conventionally dried tomato snack bars.

Due to the diversity of the bioactive compounds present in plant material, there is no single solvent capable of extracting them all. For this reason, binary systems, composed of water and organic solvent, are preferred over their pure counterparts. Water dissolves polar compounds, while the organic solvent recovers the less polar constituents. Although several organic solvents have been used to extract bioactive compounds from plant matrices, ethanol is the preferred solvent for food applications [19].

In different food matrices, such as algae containing RuBisCO, 50% ethanol dilution proved to be the best in terms of the quantity of polyphenols extracted and antioxidant power [20]. For the extraction of bioactive compounds, the bars were ground with a pestle, and 250 mg of sample was solubilized with 5 mL of 50% ethanol, homogenized with Ultraturrax (Polytron PT MR 2100, Kinematica, Switzerland), vortexed, and stirred for two hours. The samples were centrifuged at 4 °C, 2140× *g* for 10 min (centrifuge CR31, Jouan SA, Saint-herblain, France), and the supernatant was collected at 4 °C until use. Extraction was performed in triplicate.

### 2.3. Polyphenol and Flavonoid Content

The total polyphenol content was estimated as Folin–Ciocalteu reducing capacity [21]. Briefly, 100 µL of sample extract was mixed with 500 µL of Folin–Ciocalteu reagent (diluted 1:10 in water) and incubated in the dark for 5 min. Next, 400 µL of 0.7 M sodium carbonate (Na_2_CO_3_) was added. Results were expressed as mg gallic acid equivalent (GAE)/g dry weight (DW), and the absorbance was read at 760 nm using a microplate reader (FLUOstar Omega, BMG LABTECH, Ortenberg, Germany).

The flavonoid content was quantified using the aluminum chloride colorimetric method described by Kim and colleagues [22] with minor modifications. Briefly, 100 µL of the sample extracts were mixed with 400 µL of distilled water and 30 µL of 5% NaNO_2_ and incubated for 5 min at room temperature (about 25° C). Finally, 30 µL of 10% AlCl_3_ was added; following 6 min incubation, reactions were neutralized with 200 µL of 1 M NaOH and 240 µL of distilled water. Absorbance was measured after 30 min at 430 nm and the results were expressed as mg catechin equivalent (CE)/g dry weight (DW).

### 2.4. Antioxidant Activity In Vitro

The DPPH^•^ antiradical activity of the sample extract was determined according to Boudjou et al. [23]. In detail, 25 µL of opportune dilution of the sample extract was added to 975 µL of 60 µM methanolic DPPH solution. After thirty minutes in the dark, the reduction in DPPH radicals was measured at 517 nm, methanol was used as a blank, and the antiradical activity (ARA) was calculated as the percentage of DPPH^•^ inhibition using the following equation:

ARA % = [1 − (A_S_/A_C_)] × 100

where A_S_ is the absorbance of the sample and A_C_ is the absorbance of the DPPH solution. The sample extract concentration corresponding to 50% of DPPH^•^ radical inhibition (EC_50_ value, mg/mL) was calculated from the graph of ARA percentage against extract concentrations.

The oxygen radical absorbance capacity (ORAC) of the sample extracts was determined as described by Ninfali et al. [24] with modifications. An aliquot of 100 µL of sample extract or 50 µM Trolox was mixed with 800 µL of 40 nM fluorescein sodium salt in 75 mM phosphate buffer (pH 7.4) and with 100 µL of 400 mM AAPH. Fluorescein was used as a probe, and AAPH was used as a peroxyl radicals generator. Fluorescein fluorescence decay was read at λ_ex_ 485 nm and λ_em_ 510 nm. AAPH was used as a peroxyl radicals generator, fluorescein as the probe, and Trolox as the standard. Results were expressed as ORAC units (µmol Trolox Equivalents/g DW).

The ability of the sample extracts to reduce ferric iron (Fe^3+^) to ferrous iron (Fe^2+^) was evaluated by the FRAP assay according to Colosimo et al. [25] with slight modifications. Briefly, an aliquot of 33 µL of the sample extract was added to 967 µL FRAP buffer (300 mM acetate buffer pH 3.6, 10 mM TPTZ in 40 mM HCl, and 20 mM FeCl_3_·6H_2_O at a ratio of 10:1:1). After 30 min of incubation at room temperature, the absorbance was measured at 593 nm. Results were expressed as Fe^2+^ equivalents (µM), using a standard curve of FeSO_4_·7H_2_O.

### 2.5. Biofunctional Properties of Snack Bar Extracts

The pea and rubisco bars were water extracted (50 mg/mL), vortexed, and stirred for two hours. The samples were centrifuged at 4 °C, 2140× *g* for 10 min (Jouan CR3i centrifuge), and the supernatant was kept at 4 °C until use. The antidiabetic activity of the water bar extracts was evaluated by the inhibition of alpha-amylase and dipeptidyl peptidase IV (DPP4). Alpha-amylase inhibition was performed with the colorimetric alpha-amylase Inhibitor Screening Kit (ab283391, Abcam, Cambridge, UK) following the manufacturer’s instructions. The results were expressed as percentage relative inhibition using the following formula:

% Relative Inhibition = (slope of [EC]-slope of [S])/(slope of [EC]) × 100

where [EC] is the enzyme control and [S] is the test sample.

DPP-IV inhibition was performed using the DPP-IV Inhibitor Screening Assay Kit (ab133081, Abcam) following the manufacturer’s instructions. The assay uses a fluorogenic substrate, Gly Pro-Aminomethylcoumarin (AMC), to measure DPP-IV activity. Cleavage of the peptide bond by DPP releases the free AMC group, resulting in fluorescence that can be analyzed using an excitation wavelength of 355 nm and an emission wavelength of 450 nm.

The capacity of the bar extracts to inhibit the pancreatic lipase (PL), was evaluated following the protocol of Bustanji and colleagues [26]. *p*-nitrophenyl butyrate (PBNP) was used as a substrate while orlistat was used as a positive control for PL inhibition. The percentage of residual activity of PL was determined for each compound by comparing the lipase activity of PL with and without the compound against a blank using the denatured enzyme.

### 2.6. Static In Vitro Simulated Gastrointestinal Digestion and Protein and Peptide Content 

Pea and rubisco bar samples, dried either in an oven or a microwave vacuum, were digested using the INFOGEST 2.0 protocol for static in vitro gastrointestinal digestion [27]. Total protein and peptide content was determined in gastric and duodenal digested samples. Pea and rubisco bar digests (50 mg/mL) were water-extracted for 30 min at 4 °C, centrifuged for 10 min at 15,700× *g* (Eppendorf 5415R Refrigerated Centrifuge, Hamburg, Germany), and supernatants were collected and stored at 4 °C until use. The Bradford reagent was used to measure total soluble protein concentrations according to the manufacturer’s instructions, using bovine serum albumin as the standard. An aliquot of the water extracts was then treated with 50% TCA for protein precipitation, incubated for 1 h at 4 °C with shaking, and centrifuged at 15,700× *g* for 10 min, following the method of Rieder et al. [28]. After precipitation, peptide quantification was performed using the Pierce Quantitative Colorimetric Peptide Assay (Thermo Fisher Scientific, Whatman, MA, USA).

### 2.7. Human Intestinal Cell Culture

The human intestinal HT-29 cell line (DSMZ, Braunschweig, Germany), derived from human colonic adenocarcinoma, was cultured in Dulbecco’s modified Eagle’s medium/nutrient mixture F-12 (DMEM/F12) supplemented with 10% fetal bovine serum (FBS), 100 units/mL penicillin, and 100 µg/mL streptomycin purchased from Sigma-Aldrich, and kept at 37 °C with a 5% CO_2_ atmosphere until reaching 80–90% of confluence. All treatments mentioned hereafter were performed using DMEM/F12 medium without phenol red and FBS and containing antibiotics.

### 2.8. Cell Viability and Reactive Oxygen Species Determination

To assess the impact of pea and rubisco bar extracts on cell viability, HT-29 cells were seeded into a 96-well plate at a density of 10^4^ cells/well and treated with increasing concentrations of the sample (0.05, 0.5, 1, and 2 mg/mL). Following 24 h of treatment, HT-29 cells were incubated with 0.5 mg/mL MTT (thiazolyl blue tetrazolium bromide) reagent for 1 h at 37 °C in 5% CO_2_. After incubation, the cells were rinsed with PBS, released formazan crystals were dissolved in 100 μL of 10% DMSO-90% isopropanol, and the absorbance was measured at 540 nm. Data were expressed as viability % vs. the untreated cells. The concentrations identified as the highest non-cytotoxic from the assay were selected for gene expression treatments.

To quantify intracellular reactive oxygen species (ROS) levels, the cell-permeable dye 2′,7′-dichlorodihydrofluorescein diacetate (DCFH-DA) was employed. After diffusion into viable cells, DCFH-DA undergoes deacetylation by cell esterase into a nonfluorescent compound (DCFH) before being oxidized by ROS in a highly fluorescent compound, the DCF. Briefly, HT-29 (10^4^ cell/well) seeded in a fluorescence-blackened 96-well plate were exposed to 24 h of treatments with different concentrations of pea and rubisco bar extracts (0.05, 0.5, 1, and 2 mg/mL). Following exposure, cells were rinsed and incubated with 15 μM DCFH-DA for 30 min at 37 °C in the dark. ROS production was detected using the microplate reader at excitation and emission wavelengths of 485 nm and 535 nm, respectively.

### 2.9. Gene Expression Analysis with Real-Time RT-PCR

The HT-29 cells were seeded into a 6-well plate at a density of 6 × 10^5^ cells/well for gene expression analysis. After reaching confluence, the cells were pre-treated for 1 h with 0.5 mg/mL of extracts from the pea and rubisco bar samples, dried either by oven or microwave vacuum, and then stimulated for 24 h with or without 50 ng/mL of TNFα. The highest concentration of TNFα that did not reduce viability was used. After incubation, cells were collected, and the pellets were used for RNA extraction.

According to the manufacturer’s instructions, the RNA was extracted using the E.Z.N.A. Total RNA Kit I (OMEGA Bio-Tek, Norcross, GA, USA).

The total RNA obtained was then reverse transcribed using the iScript gDNA Clear cDNA Synthesis Kit (Bio-Rad, Hercules, CA, USA), according to the manufacturer’s instructions.

Real-Time RT-PCR assay was conducted in duplicate using the Bio-Rad C1000 TM thermal cycler (CFX-96 Real-Time PCR detection systems, Bio-Rad Laboratories Inc., Singapore) to determine gene expression, using SYBR Green (SsoAdvanced Universal SYBR Green Supermix, Bio-Rad Laboratories Inc., Hercules, CA, USA) for tracking cDNA amplification.

The specific primers for the COX-2 (Cyclooxigenase-2), HMOX-1 (Heme Oxygenase 1), ACTB (Beta Actin), PPIA (peptidylprolyl isomerase A), and RPL-13a (Ribosomal Protein L13a) genes were designed using OligoArchitect™ Primer and Probe Design Software (powered by Beacon Designer™, Premier Biosoft, version 2024) (Sigma-Aldrich, Saint Louis, MO, USA).

The most stably expressed reference genes (β-actin, PPIA, RPL13-a) were determined using Bio-Rad’s CFX96 manager software 3.1 (CFX-96 Real-Time PCR detection system, Bio-Rad Laboratories Inc., Hercules, CA, USA) and subsequently used to normalize mRNA expression data. Relative quantification was performed using the ΔΔCt method. The primers used in this experiment are as follows: 5′-CCGAGGTGTATGTATGAGTGT-3′ and 5′-CTGTGTTTGGAGTGGGTTTC-3′ for COX-2; 5′-GCAACAAAGTGCAAGATTCTG-3′ and 5′-GCTGAGTGTAAGGACCCAT-3′ for HMOX-1; 5′-TCTGGCACCACACCTTCT-3′ and 5′-TGATCTGGGTCATCTTCTCAC-3′ for ACTB; 5′-CTTGGGCCGCGTCTCCTTCG-3′ and 5′-TTGGGAACCGTTTGTGTTGGGGC-3′ for PPIA; and 5′-CGCCCTACGACAAGAAAAAG-3′ and 5′-CCGTAGCCTCATGAGCTGTT-3′ for RPL-13a.

### 2.10. Statistical Analysis

For statistical analysis, one-way and two-way analysis of variance (ANOVA) followed by Tukey’s multiple comparisons test were used for differences between groups, using GraphPad Prism 8.0. Values of *p* < 0.05 were considered statistically significant. Results were expressed as mean ± standard deviation (SD) of three independent experiments.

## 3. Results

### 3.1. Bioactive Content of Snack Bars

Different solvents (water, 50% acetone, 50% methanol, 50% ethanol) were tested to optimize polyphenol extraction from the snack bars. Based on preliminary findings, ethanol was selected for its efficiency in recovering bioactive compounds from both tomato- and olive-derived ingredients and for its property of being a less toxic solvent, compatible with cell tests.

The protein source markedly influenced the polyphenol content of the bars. Rubisco bars exhibited significantly higher levels of total polyphenols and flavonoids compared to pea bars, particularly when dried using microwave vacuum technology. In this condition, total phenolic content reached 10.12 ± 0.27 mg GAE/g DW, and flavonoids reached 5.65 ± 0.47 mg CE/g DW, both higher than values recorded for pea bars (Table 2).

Drying methods affected pea bars only: oven drying led to a significant increase in total polyphenol content compared to microwave vacuum drying (*p* < 0.001).

### 3.2. In Vitro Antioxidant Capacity of the Different Snack Bar Samples

The antioxidant potential of the tomato snack bars was evaluated using DPPH, ORAC, and FRAP assays (Table 3).

Comparing proteins, a distinct trend in antioxidant capacity was observed depending on the drying methods used. Antioxidant power as measured by DPPH was significantly higher in rubisco bars dried with microwave vacuum technology than in pea protein bars (*p* < 0.05), and their EC_50_ value was 1.3 times lower than the oven-dried bars. The FRAP test and the ORAC test confirmed the better antioxidant power of rubisco bars compared to the pea ones.

Considering drying methods, the ORAC assay did not show significant differences, although microwave vacuum drying resulted in higher values for rubisco bars, whereas pea bars performed slightly better after oven drying. The DPPH assay was especially sensitive to drying conditions, showing enhanced antioxidant power in rubisco bars treated with microwave vacuum drying.

### 3.3. DPP-IV and Pancreatic Lipase Inhibition

No α-amylase inhibition was observed in either pea or rubisco bars. On the contrary, both bar formulations displayed inhibitory activity against DPP-IV, with rubisco bars showing significantly stronger inhibition than their pea counterparts, independent of drying method (*p* < 0.001, Table 4).

Both microwave- and oven-treated rubisco bars had significantly lower EC_50_ values for lipase inhibition (505.8 ± 22.0 and 503.7 ± 19.2 µg/mL, respectively) than pea bars and required approximately half the concentration to achieve the same level of enzyme inhibition as pea bar extracts, indicating superior efficacy.

### 3.4. Protein and Peptide Content Following In Vitro Simulated Gastrointestinal Digestion

**Figure 1:**
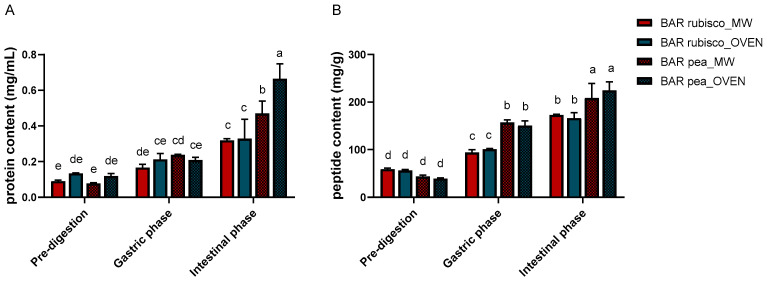
Protein (**A**) and peptide (**B**) content of pea and rubisco bar samples, processed by microwave vacuum or conventional oven drying, before and after in vitro digestion. Experiments were carried out in triplicate (n = 3) and results were expressed as mean ± SD. Different letters indicate significant differences among treatments according to two-way ANOVA followed by Tukey’s post hoc test at *p* < 0.05.

The total soluble protein and peptide content of pea and rubisco bars is presented in Figure 1A. During the gastric phase, all samples showed increased soluble protein content. Pea bars dried by oven exhibited the highest concentration (0.238 g/mL), while rubisco bars dried by microwave vacuum showed the lowest (0.167 g/mL).

In the intestinal phase, soluble protein content further increased for all samples, reflecting enhanced proteolytic activity of pancreatic enzymes and improved accessibility of protein substrates. Oven-dried pea bars showed the highest value (0.665 g/mL), followed by the microwave variant (0.470 g/mL). Differences between drying methods were minor, although microwave-treated samples showed slightly higher soluble protein content.

Peptide content followed a similar trend (Figure 1B). During the gastric phase, a significant increase in peptide levels was observed compared to undigested samples, particularly in the pea bars. Also, in the intestinal phase, pea bars showed the highest peptide concentrations, indicating a more efficient breakdown of proteins into smaller fragments. This suggests a higher degree of digestibility and potential for better absorption of bioactive peptides in these samples. Rubisco bars, while also showing increased peptide content post-intestinal digestion, had overall lower values than the pea bars.

### 3.5. Cell Viability and Intracellular ROS Levels in HT-29 Cells

**Figure 2:**
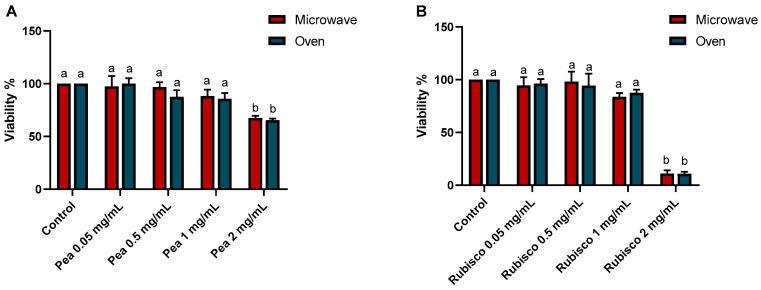
MTT assay on HT-29 cells after 24 h of incubation with or without (control) increasing concentrations of pea (**A**) and rubisco (**B**) bar extracts (0.05, 0.5, 1, and 2 mg/mL). Data were expressed as viability % vs. the untreated cells. Experiments were carried out in triplicate and results were expressed as mean values ± SD. Different letters indicate significant differences among treatments, according to two-way ANOVA followed by Tukey’s test at *p* < 0.05.

The MTT assay was performed on HT-29 cells to test the cytotoxicity and biocompatibility of pea (Figure 2A) and rubisco (Figure 2B) snack bars, dried by microwave vacuum and oven, after 24 h of treatments. Increasing concentrations (0.05, 0.5, 1, and 2 mg/mL) of both pea and rubisco bar extracts showed a dose-dependent decrease in viability. The concentration tested was chosen to cover a broad spectrum of potential exposure levels, as no specific references on snack bar extracts are available in the literature. The concentration of 2 mg/mL of both pea and rubisco bar shows a significant cytotoxic effect, with a viability reduction of 40% and 80%, respectively (*p* < 0.001). This supraphysiological value is not compatible with the bioavailability or circulating levels expected after normal consumption but allows us to define a maximum limit for safety evaluation.

**Figure 3:**
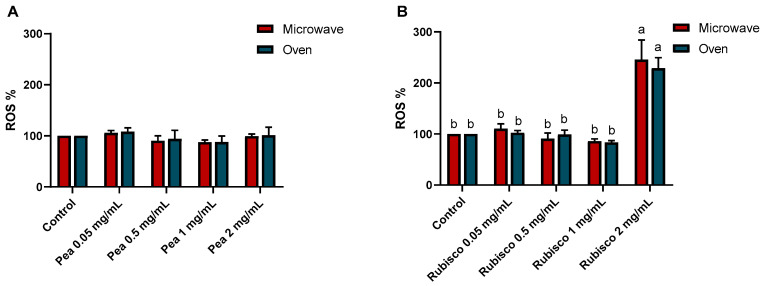
ROS levels after 24 h of treatments with or without increasing concentrations of pea (**A**) and rubisco (**B**) bar extracts (0.05, 0.5, 1, and 2 mg/mL). The results were expressed as the DCF fluorescence level with respect to untreated cells (0 mg/mL). Experiments were carried out in triplicate and results were expressed as mean values ± SD. Different letters indicate significant differences among treatments, according to two-way ANOVA followed by Tukey’s multiple comparisons test at *p* < 0.05.

Furthermore, to assess the antioxidant properties of the tomato bars, the intracellular generation of reactive oxygen species (ROS) in HT-29 cells was measured using 2,7-dichlorofluorescein staining (Figure 3). After 24 h of treatment with different concentrations of pea bar extracts, ROS generation showed no significant differences in pea bar extracts compared to controls. In contrast, the rubisco bar extract at higher concentrations led to a significant increase in ROS levels compared to control cells (*p* < 0.001).

### 3.6. Anti-Inflammatory Effects of Snack Bars on HT-29 Cells

**Figure 4:**
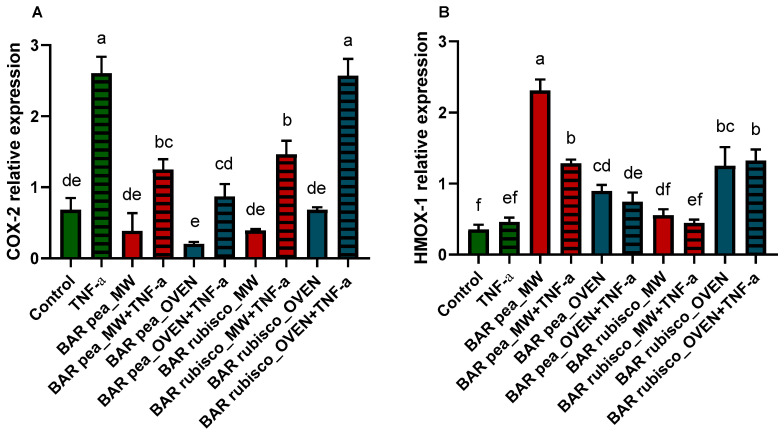
Quantitative RT-PCR analysis of COX-2 (**A**) and HMOX-1 (**B**) gene expression in HT-29 1 h pretreated with or without 0.5 mg/mL pea and rubisco bar extract, dried by microwave (MW) vacuum or conventional oven, then exposed 24 h to 50 ng/mL TNF-α. Experiments were carried out in triplicate. Different letters indicate significant differences among treatments, according to one-way ANOVA and Tukey’s multiple comparisons test at *p* < 0.05.

To study the potential anti-inflammatory effects of the bar extracts, we evaluated the expression of COX-2—a key enzyme elevated in inflammatory bowel disease—and HMOX-1—which exerts cytoprotective effects by inhibiting NF-κB signaling and reducing oxidative stress— and the results are represented in Figure 4. The HT-29 cells were treated with 0.5 mg/mL of pea and rubisco bar extracts; moreover, cells were pretreated with 0.5 mg/mL of extract and stimulated with 50 ng/mL of TNF-α for 24 h to induce an inflammatory state. COX-2 expression was significantly higher in TNFα-treated cells compared to controls. Additionally, exposure to 0.5 mg/mL of either microwave-vacuum-dried or oven-dried pea bar extracts did not alter basal COX-2 levels, with no notable difference between the two treatments. Conversely, pretreatment with the pea bar extracts significantly reduced TNFα-induced COX-2 upregulation, bringing levels close to those of the control, particularly in the case of the oven-dried pea bar. In contrast, rubisco bar treatment did not affect basal COX-2 levels. However, unlike pea bars, the oven-dried rubisco bars failed to reduce TNFα-induced COX-2 upregulation.

Pea bar extracts increased HMOX-1 expression compared to control and rubisco bar treated cells, with microwave-dried bars showing the highest levels. However, pretreatment followed by TNF-α exposure led to a reduction in HMOX-1 expression compared to cells treated with pea bar extracts alone. Whereas, after the rubisco bar extract treatment, HMOX-1 expression increased only in response to the oven-dried treatment, with no differences observed after TNF-α exposure (Figure 4).

## 4. Discussion

Compared to pea protein, RuBisCO consistently enhanced the functional properties of tomato snack bars. Higher polyphenol and flavonoid contents in rubisco bars suggest that protein type can modulate the retention and stability of bioactive compounds during formulation and drying [29,30].

The influence of drying method varies according to protein type. In pea bars, oven drying enhanced polyphenol release, likely due to thermal breakdown of cell walls, as previously observed, which promotes the release of bound phenolics or their conversion into more soluble forms [31,32]. In rubisco bars, microwave vacuum drying proved advantageous, aligning with studies showing that mild thermal treatments can enhance antioxidant activity without extensive degradation [33]. For instance, Torres et al. [34] observed that heat increases the levels of specific compounds like *p*-coumaric acid and quercetin, while reducing others such as gallic acid, reflecting selective changes in the polyphenolic profile.

Antioxidants can prevent or reduce damage from free radicals, such as lipid peroxidation, oxidative damage to cell membranes, protein glycation, and enzyme inactivation. In the food and beverage industry, they extend the shelf life of products by safeguarding them against oxidation, which can lead to fat rancidity and color changes [35,36]. Given their dual importance, the antioxidant potential of the tomato snack bars was evaluated using a multi-assay approach (DPPH, ORAC, and FRAP) to capture different mechanisms of antioxidant action. As emphasized by López-Alarcón and Denicola [37], combining multiple tests provides a more comprehensive and reliable assessment of antioxidant properties. Antioxidant assays confirmed rubisco bars to be superior, especially after microwave vacuum drying. This observation is consistent with Kobbi et al. [38], who demonstrated potent antioxidant properties of RuBisCO hydrolysates, attributed to sulfur-containing amino acids such as cysteine and methionine. These amino acids, which are considered critically low in meat-reduced diets, are present in high concentrations in RuBisCO [16,39]. The different behavior, upon drying, of the two different types of proteins may depend on the thermal degradation, which decreases the concentration of phytochemicals, and, on the other hand, on the matrix softening that facilitates the extraction of phytochemicals, resulting in a higher concentration compared to the raw material [40]. Moreover, the covalent bond of polyphenols to the protein, with the introduction of hydroxyl groups in the structure, contributes to the higher antioxidant capacity of proteins [41]. Overall, the results highlight the superior antioxidant potential of rubisco snack bars, particularly when processed via microwave vacuum drying. These findings underscore the synergistic influence of protein type and mild drying techniques in preserving and enhancing the functional quality of plant-based snack formulations.

Bioactivity assays further highlighted the functional advantage of rubisco bars. Their stronger inhibition of DPP-IV and pancreatic lipase indicates potential for regulating postprandial glycemia and lipid absorption, key targets in the prevention of diabetes and obesity [42,43,44]. The stability of these effects across drying methods suggests robust functionality. The inhibition of pancreatic lipase represents a promising strategy for obesity management, as it reduces the intestinal absorption of dietary fats, thereby lowering caloric intake and promoting weight control [45]. Currently, orlistat is the only clinically approved pancreatic lipase inhibitor; however, the search for safe plant-derived alternatives has gained considerable attention. Natural compounds capable of modulating lipid metabolism through pancreatic lipase inhibition may provide an effective and safer basis for the development of anti-obesity functional foods or nutraceuticals, with fewer side effects compared to conventional drugs [46].

In contrast, digestibility tests revealed that pea bars released more soluble protein and peptides during simulated digestion. This differs from reports on rubisco’s high digestibility [47,48], possibly due to matrix effects or polyphenol–protein interactions that limited accessibility. These findings highlight the need for further investigation of food matrix effects on rubisco digestibility.

The MTT assay on intestinal cells represents a method for assessing the biocompatibility and safety of food formulations. Given the current lack of data on the cytotoxicity of tomato-based snack bar extracts, a range of extract concentrations (0.05–2 mg/mL) was tested to cover both physiologically relevant doses and higher limits, providing insight into potential dose-dependent effects. Rubisco and pea bar extracts both reduced cell viability at the highest concentration tested, possibly due to lycopene contributions [49] with a strong decrease in rubisco extract. This increased cytotoxic effect of the rubisco bar does not seem to be caused by the protein itself. Hurley and colleagues [50], after treating monolayers of human gut epithelial cells with concentrations of up to 2 mg/mL of bovine serum albumin, bovine fibronectin, and spinach RuBisCO found the absence of any negative effect on cell viability (reduction in LDH and MTT) or barrier integrity. Notably, rubisco bar extracts exhibited an increase in intracellular ROS at the highest concentration. This rise in ROS may explain the cytotoxic effects observed at the same concentration in the MTT assay. Superoxide accumulation triggers the activation of AMP-activated protein kinase (AMPK), which subsequently upregulates transcription factors, promoting the expression of pro-apoptotic genes [51]. The discrepancy observed between the high antioxidant activity of rubisco bars in chemical assays and the pro-oxidant effects observed in cells at high concentration may be attributed to the higher polyphenols content detected in the rubisco bar extracts, with respect to the pea bars. Phenolic compounds can either prevent or promote the oxidation process, depending on environmental conditions such as pH, the presence of metal ions, and their concentrations, directly affecting cell viability [52]. Furthermore, certain flavonoids, under cellular conditions, can interact with thiol and amino groups of proteins, leading to the formation of protein adducts and oxidative modifications [53]. This mechanism could partly explain why rubisco snack bars, despite exhibiting strong antioxidant capacity in chemical assays, induced ROS generation in cells at the highest tested concentration. It is therefore plausible that certain phenolic compounds or peptides released from rubisco bars may undergo redox cycling or quinone formation, acting as pro-oxidants in cellular environments [54]. For these reasons, a more detailed characterization of the bioactive compounds released from the two snack bars should be undertaken in further studies.

Tumor necrosis factor alpha (TNF-α) is a cytokine that drives inflammatory responses by inducing inflammatory gene expression and, indirectly, cell death, promoting inflammatory immune reactions. The binding of TNF-α to a TNF receptor 1 (TNFR1) leads to the activation of the nuclear factor-kB (NF-kB) pathway resulting in the transcription of proinflammatory genes and the increase in cyclooxygenase 2 expression [55,56,57]. COX-2 is one of the main proinflammatory enzymes and is selectively expressed in response to various inflammatory stimuli and is upregulated in inflammatory bowel disease [58]. Many papers demonstrate that nuclear translocation of NF-kB was inhibited through the production of end products (bilirubin, CO) with heme oxygenase-1 (HO-1) activity, with anti-inflammatory effect [59,60]. HO-1 and its products exert beneficial effects by preventing oxidative damage, regulating apoptosis, and modulating inflammatory responses. The nuclear factor erythroid-2 related factor 2 (Nrf2)/heme oxygenase-1 pathway plays an important role in the occurrence and regulation of oxidative stress and inflammatory responses [61]. The anti-inflammatory assays revealed that pea bars were more effective in reducing TNFα-induced COX-2 expression and enhancing HMOX-1 expression, suggesting a protective role in gut inflammation. Rubisco bars, despite their higher polyphenol and antioxidant contents, did not confer the same benefit in cell lines studies, suggesting that protein type differentially modulates inflammatory pathways.

Overall, rubisco snack bars exhibited superior antioxidant and enzyme-inhibitory activities, whereas pea-based bars showed advantages in protein digestibility and anti-inflammatory effects. Future research should focus on clarifying the interactions between protein structure, phenolic compounds, and processing methods, as well as validating these in vitro results in human intervention studies.

## 5. Conclusions

The present study highlights the potential of tomato-based snack bars formulated with RuBisCO and pea proteins as promising functional food products. The results reveal that protein source and drying method substantially influence the bioactive profile and functional properties of the bars. Rubisco bars exhibited higher levels of polyphenols and flavonoids, leading to superior antioxidant activity, and demonstrated significantly greater inhibition of DPP-IV and pancreatic lipase enzymes compared to pea-based formulations, thereby suggesting a potential role in modulating glycaemic response and lipid metabolism. In contrast, pea bars showed enhanced anti-inflammatory activity, particularly through the downregulation of TNF-α-induced COX-2 expression and the upregulation of HMOX-1, indicating modulation of oxidative and inflammatory pathways. Additionally, pea protein bars exhibited greater digestibility and peptide release during simulated gastrointestinal digestion. Regarding processing, microwave vacuum and conventional oven drying methods effectively preserved the functional attributes of the bars, with microwave treatment offering modest improvements in antioxidant retention and digestibility. Although these in vitro findings provide insight into the functional potential of plant-protein-enriched bars, further studies on consumer acceptance and health benefits are needed. In particular, clinical trials should be conducted to evaluate physiologically relevant doses and to confirm their impact on glycaemia regulation, lipid metabolism, oxidative stress, and inflammation in humans.

## Figures and Tables

**Table 1:** Ingredients and process of creating tomato bar samples.

	Sample			
Ingredients	1	2	3	4
Tomato (g)	100	100	100	100
Pectin type	LMP	LMP	LMP	LMP
Pectin amount (g)	1	1	1	1
Protein type	PEA	RUBISCO	PEA	RUBISCO
Protein amount (g)	10	1	10	1
Salt (g)	2	2	2	2
Tomato powder (g)	5	9.5	5	9.5
Tomato peel powder (g)	5	9.5	5	9.5
Olive powder (g)	2	2	2	2
Basil (g)	1	1	1	1
Thyme (g)	1	1	1	1
Red pepper (g)	1	1	1	1
Total weight (g)	128	128	128	128
Process Parameters				
MW power * (%)	60	60	-	-
Drying duration (min)	10	10	-	-
Vacuum pressure (Torr)	380	380	-	-
Oven temperature (°C)	-	-	120	120
Drying duration (min)	-	-	90	90

* Total microwave power (100%) is 2 kW, 2450 MHz.

**Table 2:** Bioactive content of the tomato snack bars. Different letters within each column indicate significant differences according to one-way ANOVA with Tukey’s multiple comparisons test; *p*-values < 0.05 were considered statistically significant.

	Samples	Polyphenols (mg GAE/g DW)	Flavonoids (mg CE/g DW)
Microwave	Pea bar	7.00 ± 0.17 ^c^	3.61 ± 0.45 ^b^
	Rubisco bar	10.12 ± 0.27 ^a^	5.61 ± 0,47 ^a^
Oven	Pea bar	9.01 ± 0.50 ^b^	3.94 ± 0.54 ^b^
	Rubisco bar	9.94 ± 0.41 ^a^	5.65 ± 0.42 ^a^

**Table 3:** DPPH, ORAC, and FRAP assays of tomato snack bars. Different letters within each column indicate significant differences according to one-way ANOVA with Tukey’s multiple comparisons test; *p* values < 0.05 were considered statistically significant.

	Samples	DPPH (EC_50_ mg/mL)	ORAC (µmol TE/g DW)	FRAP (µM Fe^2+^)
Microwave	Pea bar	0.67 ± 0.09 ^b^	184.16 ± 15.44 ^b^	3291.56 ± 233.75 ^b^
	Rubisco bar	0.46 ± 0.09 ^a^	226.80 ± 22.42 ^a^	4561.25 ± 94.77 ^a^
Oven	Pea bar	0.63 ± 0.06 ^b^	198.86 ± 20.87 ^ab^	3877.50 ± 221.31 ^ab^
	Rubisco bar	0.60 ± 0.09 ^ab^	202.25 ± 11.47 ^ab^	4549.38 ± 584.96 ^a^

**Table 4:** DPP-IV and pancreatic lipase inhibitory activity of microwave vacuum- and oven-dried pea and rubisco bars. Different letters within each column indicate significant differences according to one-way ANOVA with Tukey’s multiple comparisons test; *p* values < 0.05 were considered statistically significant.

	Samples	DPP-IV (% Inhibition)	Lipase (EC_50_ µg/mL)
Microwave	Pea bar	16.3 ± 2.8 ^b^	1238.7 ± 99.7 ^c^
	Rubisco bar	44.3 ± 3.9 ^a^	505.8 ± 22.0 ^a^
Oven	Pea bar	14.1 ± 0.4 ^b^	1012.7 ± 64.3 ^b^
	Rubisco bar	46.8 ± 1.2 ^a^	503.7 ± 19.2 ^a^

## Data Availability

The raw data supporting the conclusions of this article will be made available by the authors on request.
